# Supplementary material for: Genetically- and spatially-defined basolateral amygdala neurons control food consumption and social interaction
Source: Nat Commun. 2024 Aug 11;15:6868. doi: 10.1038/s41467-024-50889-7 (PMC11316773; doi:10.1038/s41467-024-50889-7)
Supplement: Supplementary file 1 — Supplementary Information [file 41467_2024_50889_MOESM1_ESM.pdf]

Supplementary Information

Genetically- and spatially-defined basolateral amygdala neurons control food consumption and social interaction

Hansol Lim[1], Yue Zhang[2], Christian Peters[1], Tobias Straub[3], Johanna Luise Mayer [1], Rüdiger Klein[1*]

1. Department Molecules – Signaling – Development, Max Planck Institute for Biological Intelligence, Martinsried, Germany

2. Department Synapses – Circuits - Plasticity, Max Planck Institute for Biological Intelligence, Martinsried, Germany

3. Biomedical Center Core Facility Bioinformatics, LMU, Munich, Germany

*Correspondence: ruediger.klein@bi.mpg.de (R.K.)

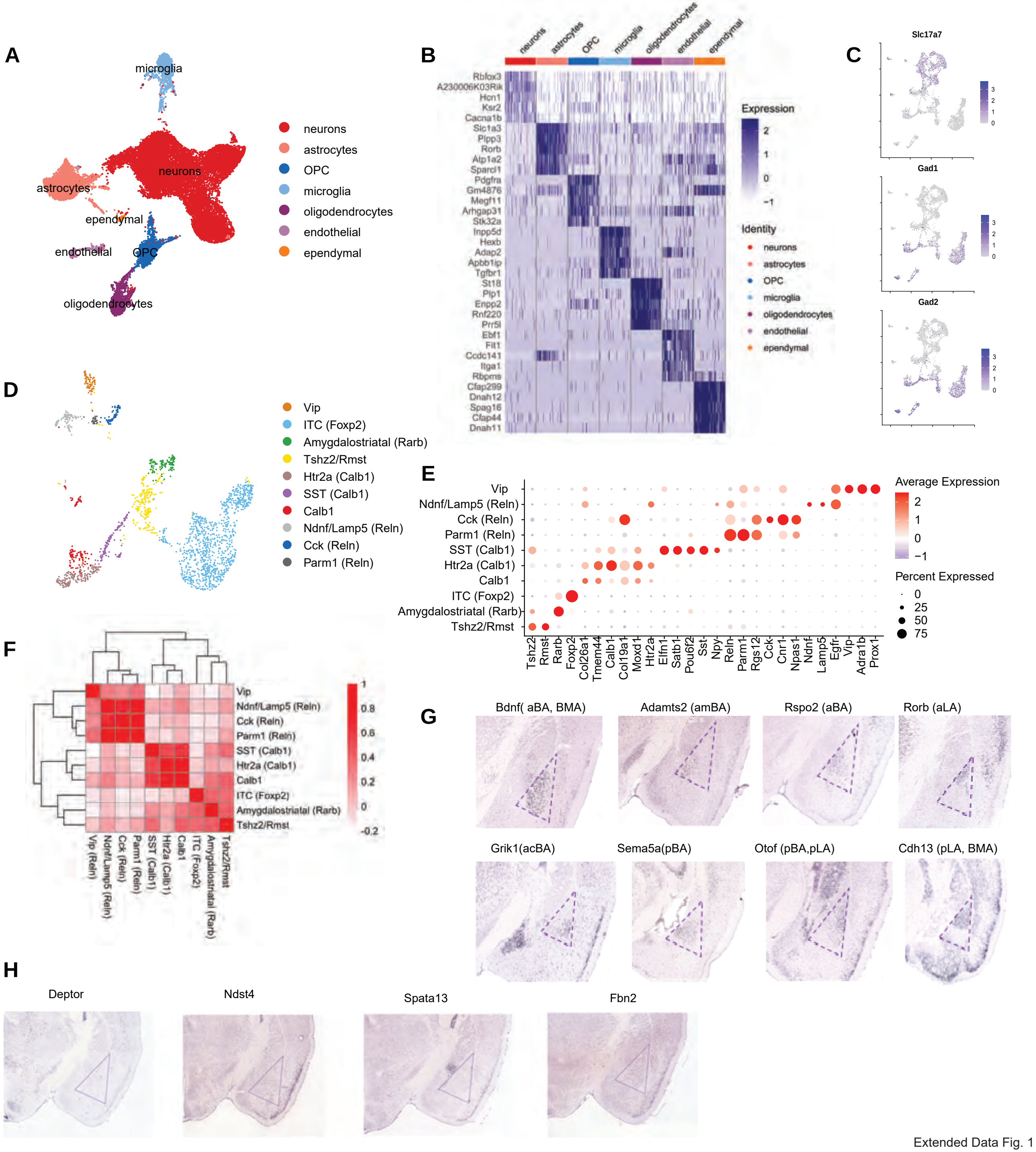

Extended Data Fig. 1

Supplementary Fig. 1 related to Figure 1: GABAergic cell clusters in BLA and marker gene selection for glutamatergic cell clusters.

(A) UMAP of all BLA cells with different cell types identified by established markers.
(B) Heatmap illustrating expression levels of top 5 marker genes in different cell types.
(C) Expression of neurons with glutamatergic (Slc17a7) and GABAergic markers (Gad1, Gad2).
(D) UMAP of GABAergic neurons with annotated clusters.
(E) Molecular signatures of GABAergic neuronal clusters based on expression of selected marker genes.
(F) Heatmap visualization of pairwise correlation matrix of marker gene expression between clusters of GABAergic BLA neurons. The scale bar indicates Pearson's R.
(G) Representative ISH images from Allen ISH data for some of the final 10 marker genes for glutamatergic clusters.
(H) Representative ISH images from Allen ISH data for genes not selected as marker genes due to their widespread expression in many brain regions (Ndst4, Fbn2), low expression (Deptor), or high expression in other brain regions (Spata13).

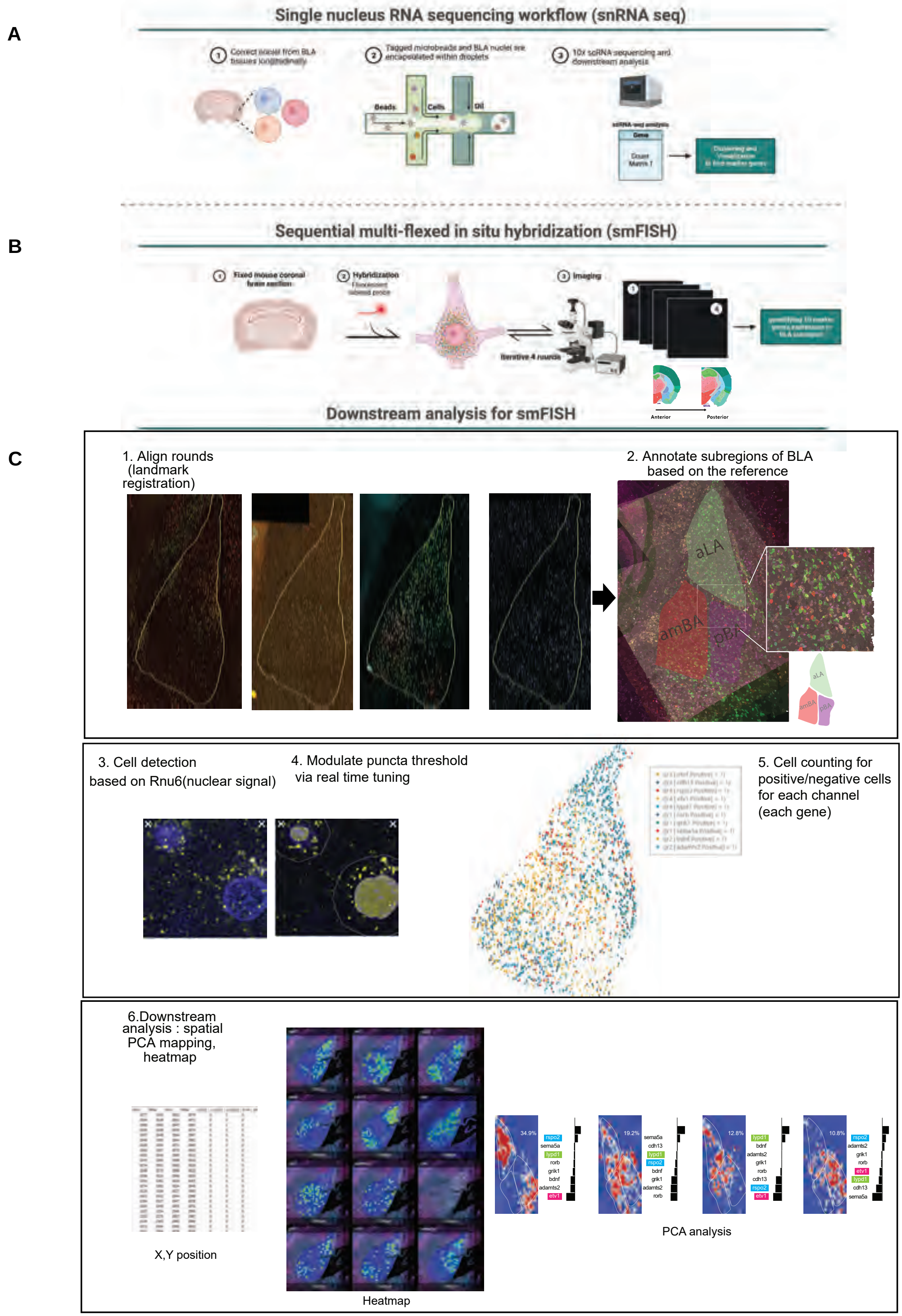

Extended Data Fig. 2

Supplementary Fig. 2 related to Figure 1-2: Workflow of snRNAseq and smFISH.

Whole Workflow overview from snRNA seq to smFISH image analysis.

(A) Nuclei from BLA tissues were harvested and processed through 10x genomics scRNAseq tools.

(B) Fixed BLA coronal sections were iteratively hybridized and imaged with 10 marker gene probes with different fluorescence.

(A) and (B) were created with BioRender.com released under a Creative Commons Attribution-NonCommercial-NoDerivs 4.0 International license

(C) Image analysis: Four images from 4 rounds of hybridization were superimposed by landmark and serial strain registration, and subregions in BLA were delineated by reference [1]. Cell segmentation (based on nuclear Rnu6 expression) was created by the HALO software with a given parameter (8 μm around the nucleus to target 12 μm of cell diameter). This follows the assumption that an average BLA glutamatergic neuron has a diameter of 10-13 μm [2,3]. The thresholding fluorescence for positive cells for each gene was performed and quantified using HALO software. After preprocessing, the dataset including the x,y positions of positive cells was transferred to the Python workspace for downstream analysis (e.g., PCA and correlation analysis). Details are explained in Methods.

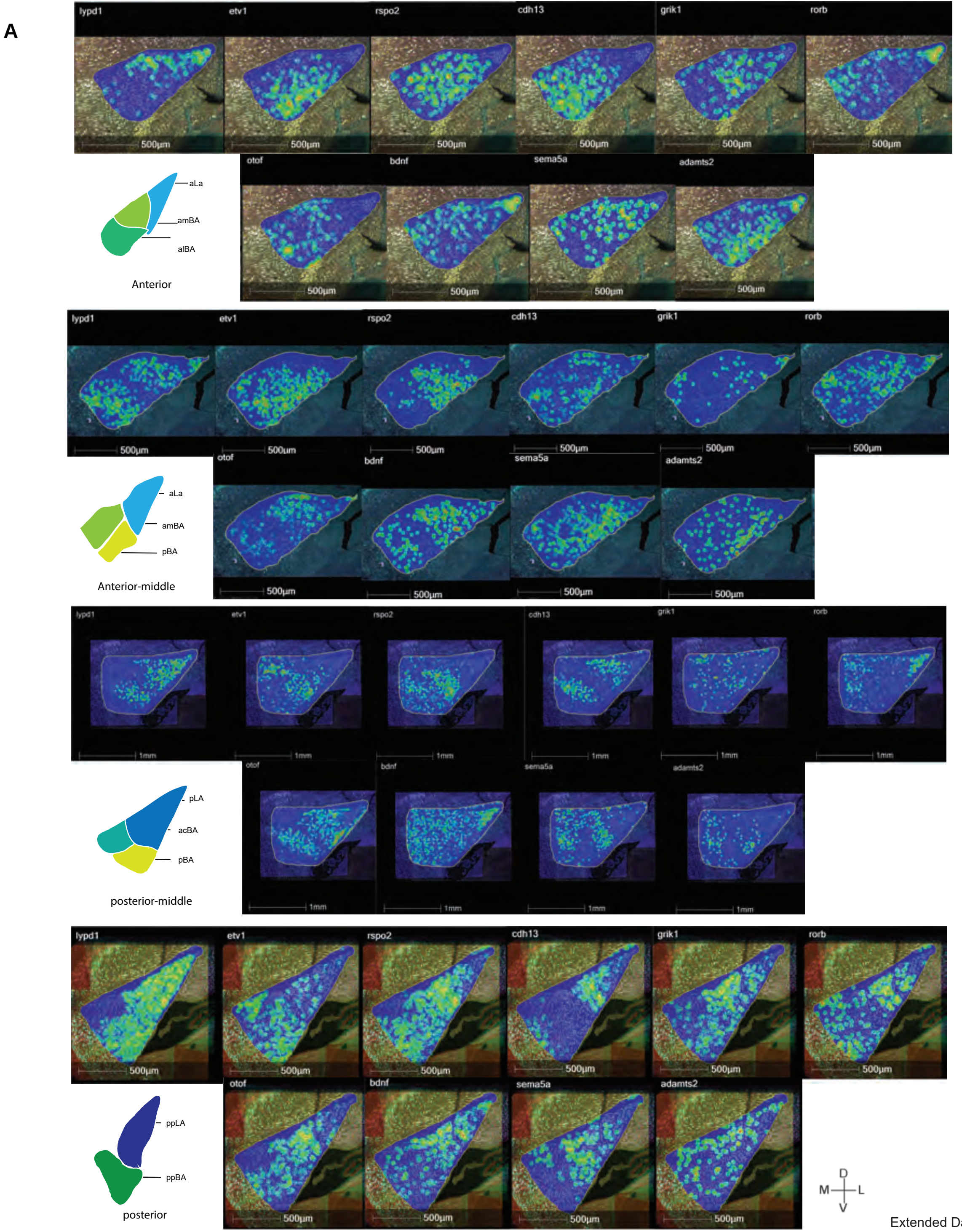

Extended Data Fig. 3

Supplementary Fig. 3 Related to Figure 2B: Representative images showing positive cells for each marker gene in BLA.

(A) Density heatmaps of selected gene expressions in BLA. Representative samples of 10 marker genes at 4 positions are shown from anterior (left top) to posterior (right bottom). The colors represent expression levels, from blue to red color indicating the lowest and the highest expression levels (radius: 25um). Scale bar: 500μm, M: medial, L: lateral, D: dorsal, V: ventral.

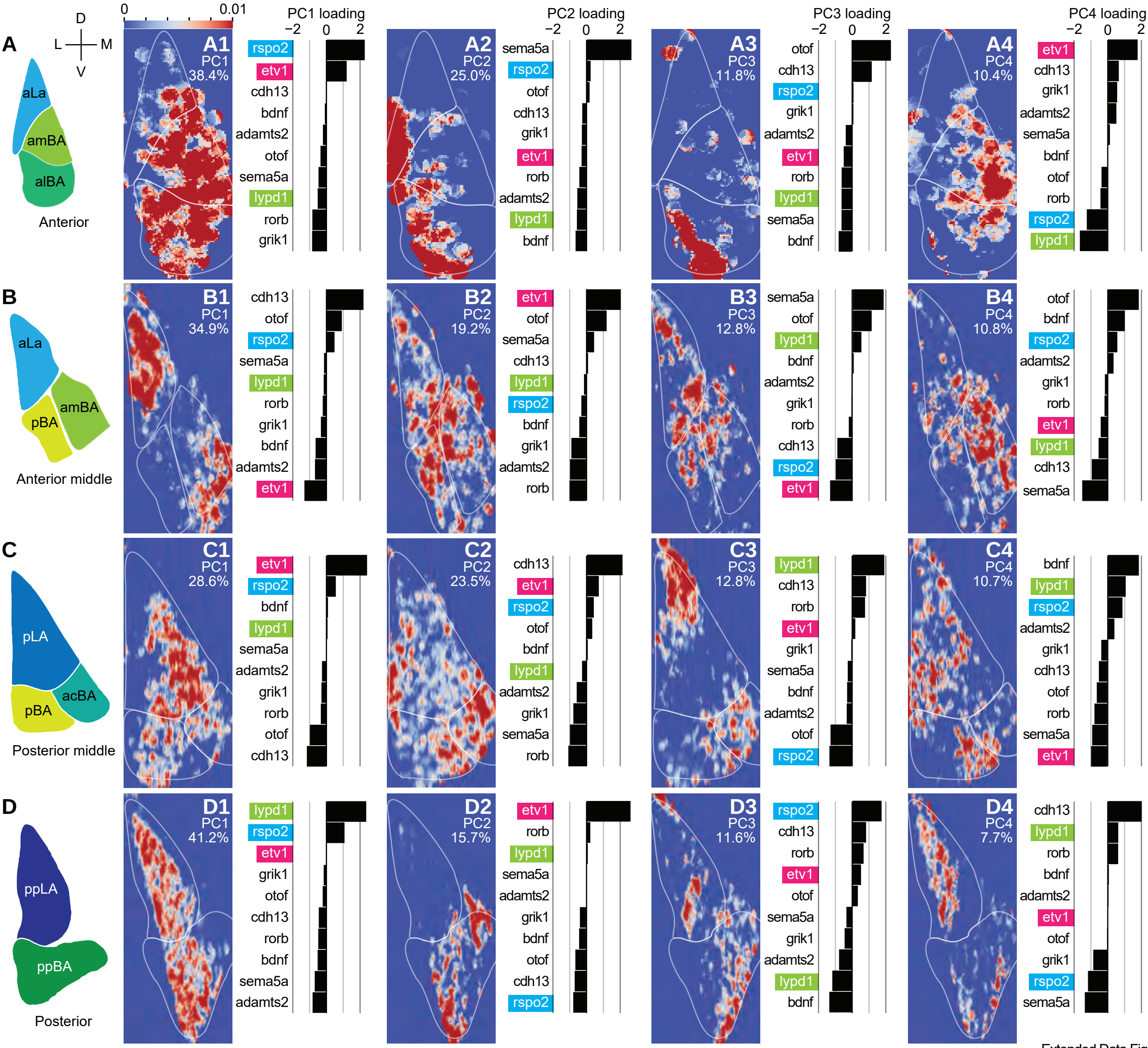

Extended Data Fig. 4

Supplementary Fig. 4 related to Figure 2B: The combination of marker genes predicts the spatial localization in the BLA.

(A-D) Left: Anatomical parcellations from anterior (A), anterior-middle (B), posterior-middle (C), and posterior (D) BLA as previously published [1]. Right: Eigen-images from the top 4 principal components (PCs) in 4 samples with the percentage of each PC from the spatial distribution of principal component analysis (PCA) of marker-genes in single cell resolutions with each bar graph of PC loading values from each marker gene for each PC axis; PC1 to PC4 (left to right in order). Contributions of Rspo2, Etv1, and Lypd1-positive cells to LA- and BA-specific PCs are highlighted.

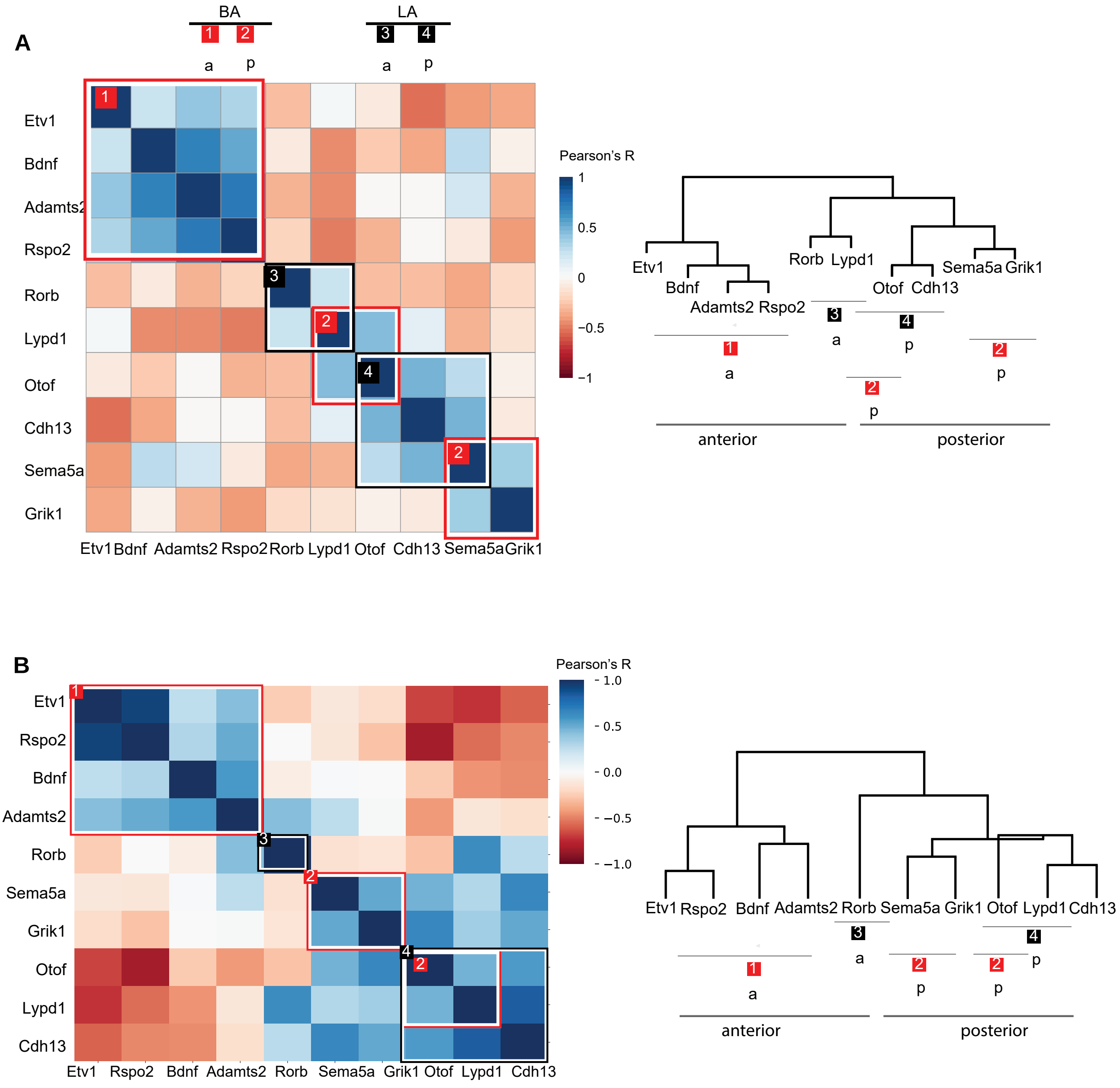
A
BA
LA
1
2
3
4
a
p
a
p
Etv1
Bdnf
Adamts2
Rspo2
Rorb
Lypd1
Otof
Cdh13
Sema5a
Grik1
Pearson's R
1
0.5
0
−0.5
−1
anterior
posterior
B
Pearson's R
1.0
0.5
0.0
−0.5
−1.0
Etv1
Rspo2
Bdnf
Adamts2
Rorb
Sema5a
Grik1
Otof
Lypd1
Cdh13

Supplementary Fig. 5 related Figures 1-2: Correlation patterns from snRNAseq and smFISH data.

(A) Pairwise correlation heatmap with hierarchical clustering of marker gene expression across all glutamatergic neurons (snRNAseq data, related to Figure 1) Scale bar indicates Pearson's correlation coefficient.

(B) Pairwise correlation heatmap with hierarchical clustering of marker gene expression across eight BLA subregions (smFISH data, related to Figure 2); Boxes in heatmap and lines in dendrogram: manual grouping of closely correlated genes by their spatial expression, red for basal (BA), black for lateral (LA) and 1, 3 for anterior (a) and 2,4 for posterior (p).

A

cluster\marker-genes	Bdnf	Adamts2	Rorb	Grik1	Sema5a	Rspo2	Etv1	Lypd1	Otof	Cdh13
cl1: Rspo2/Etv1/Adamts2/Bdnf	1	1	0.39	0.36	0.75	1	1	0.74	0.63	0.56
cl2: Sema5a/Otof/Lypd1/Cdh13	0.48	0.61	0.37	1	0.96	0.47	0.63	1	1	1
cl3: Otof	0.86	0.8	0.11	0.74	0.96	0.77	0.86	0.79	0.99	0.77
cl4: Etv1	0.77	0.62	0.71	0.22	0.19	0.68	0.92	0.84	0.57	0.56
cl5: Sema5a	0.89	0.8	0.29	0.93	1	0.43	0.35	0.47	0.76	0.86
cl6: Otof/Cdh13	0.42	0.79	0.47	0.63	0.55	0.58	0.67	0.88	0.99	0.96
cl7: Rorb	0.42	0.43	0.87	0.45	0.46	0.58	0.55	0.58	0.61	0.6
cl8: Rorb/Lypd1	0.52	0	1	0.24	0	0.46	0.37	0.82	0.21	0.6
cl9: Etv1/Lypd1	0.07	0.57	0.05	0.24	0.46	0.35	0.67	0.69	0.55	0.68
cl11: Rspo2	0.21	0.58	0.29	0	0.46	0.69	0	0	0.06	0.74
cl12: Grik1	0	0.11	0	0.73	0.15	0	0.31	0.27	0	0

B

	Bdnf	Adamts2	Rorb	Grik1	Sema5a	Rspo2	Etv1	Lypd1	Otof	Cdh13		R Cell #3234	R Cell #1936	R Cell #5065
cl1: Rspo2/Etv1/Adamts2/Bdnf	1.00	1.00	0.39	0.36	0.75	1.00	1.00	0.74	0.63	0.56		0.36	0.36	-0.20
cl2: Sema5a/Otof/Lypd1/Cdh13	0.48	0.61	0.37	1.00	0.96	0.47	0.63	1.00	1.00	1.00		-0.16	-0.38	0.71
cl3: Otof	0.86	0.80	0.11	0.74	0.96	0.77	0.86	0.79	0.99	0.77		0.14	0.01	0.29
cl4: Etv1	0.77	0.62	0.71	0.22	0.19	0.68	0.92	0.84	0.57	0.56		0.46	0.11	-0.11
cl5: Sema5a	0.89	0.80	0.29	0.93	1.00	0.43	0.35	0.47	0.76	0.86		-0.44	-0.33	0.19
cl6: Otof/Cdh13	0.42	0.79	0.47	0.63	0.55	0.58	0.67	0.88	0.99	0.96		-0.04	-0.20	0.63
cl7: Rorb	0.42	0.43	0.87	0.45	0.46	0.58	0.55	0.58	0.61	0.60		-0.01	0.07	0.08
cl8: Rorb/Lypd1	0.52	0.00	1.00	0.24	0.00	0.46	0.37	0.82	0.21	0.60		-0.06	0.04	0.16
cl9: Etv1/Lypd1	0.07	0.57	0.05	0.24	0.46	0.35	0.67	0.69	0.55	0.68		0.34	-0.12	0.61
cl11: Rspo2	0.21	0.58	0.29	0.00	0.46	0.69	0.00	0.00	0.06	0.74		-0.36	0.46	0.16
cl12: Grik1	0.00	0.11	0.00	0.73	0.15	0.00	0.31	0.27	0.00	0.00		0.23	-0.24	-0.17
Cell #3234	0.00	0.00	0.00	0.00	0.00	0.00	0.33	0.00	0.00	0.00		cl4: Etv1		
Cell #1936	0.00	0.00	0.00	0.00	0.00	0.14	0.00	0.00	0.00	0.00			cl11: Rspo2	
Cell #5065	0.00	0.00	0.00	0.00	0.10	0.02	0.00	0.15	0.07	0.15				cl2:Sema5a/Otof/Lypd1/Cdh13

C

mFISHgene expression/Cell ID	#1936	#3234	#5065
Bdnf	0	0	0
Adamts2	0	0	0
Rorb	0	0	0
Grik1	0	0	0
Sema5a	0	0	0.097561
Rspo2	0.142857	0	0.02439
Etv1	0	0.333333	0
Lypd1	0	0	0.146341
Otof	0	0	0.073171
Cdh13	0	0	0.146341
cluster ID			
cl4: Etv1	0	1	0
cl9: Etv1/Lypd1	0	0	0
cl12: Grik1	0	0	0
cl3: Otof	0	0	0
cl6: Otof/Cdh13	0	0	0
cl7: Rorb	0	0	0
cl8: Rorb/Lypd1	0	0	0
cl11: Rspo2	1	0	0
cl1 : Rspo2/Etv1/Adamts2/Bdnf	0	0	0
cl5: Sema5a	0	0	0
cl2: Sema5a/Otof/Lypd1/Cdh1	0	0	1
X max	2006	1813	1100
Y max	1775	2175	666

Supplementary Fig. 6 related to Figure 3: Workflow to anchor transcriptional clusters directly onto BLA space.

(A) The scaled sum of the expression of ten marker genes in all cells per cluster from transcriptomics.

(B) Representative data tables explaining how to calculate the correlation coefficient (R) from a single smFISH cell (# 3234 for cl4, # 1936 for cl11, # 5065 for cl2) to assign the best correlated transcriptional cluster.

(C) Representative table showing the assignment of three example cells from smFISH to the best correlated transcriptional cluster and the corresponding X, Y position in the BLA.

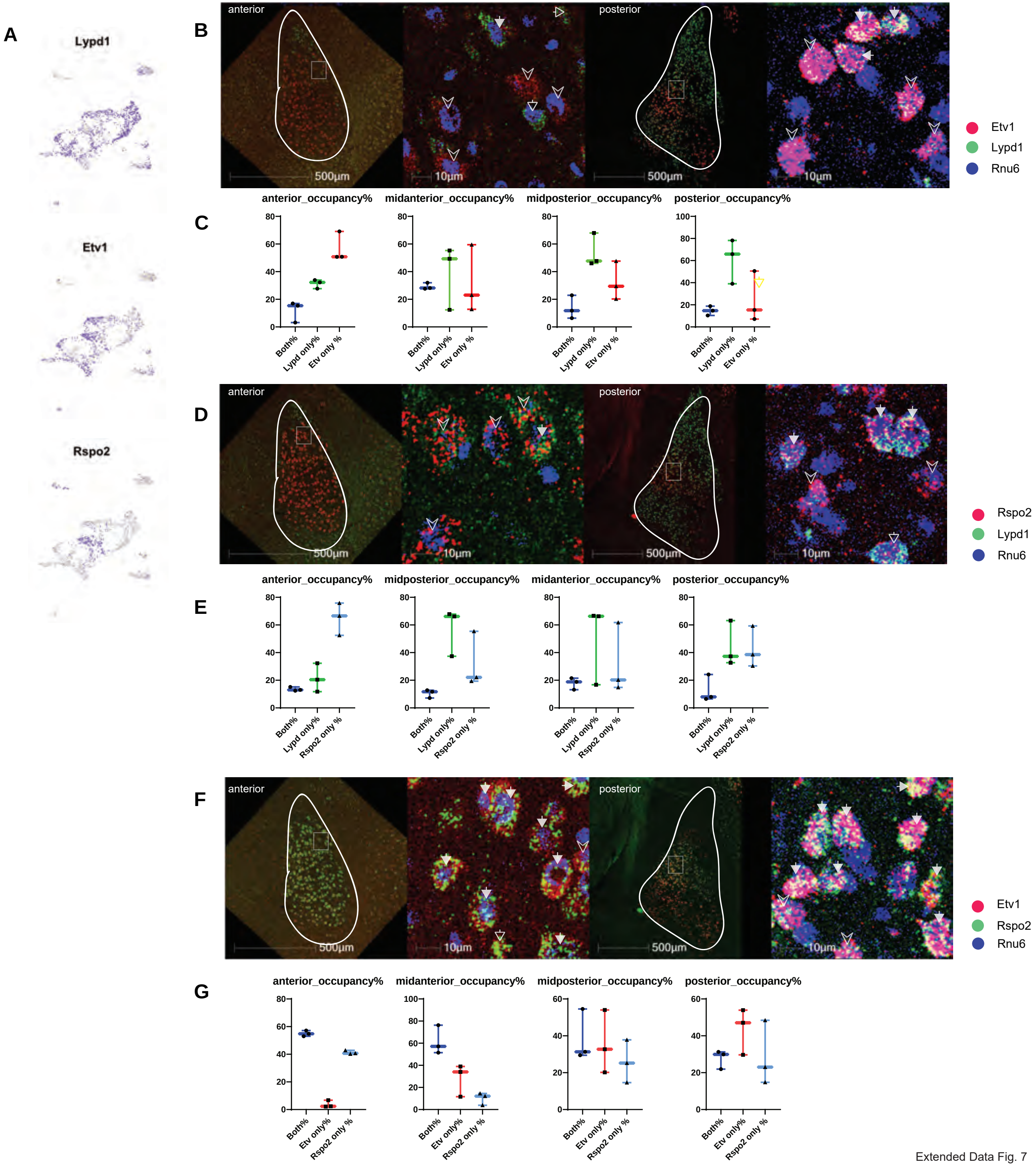

Extended Data Fig. 7

Supplementary Fig. 7 related to Figure 4-7: Comparison of *Lypd1-, Rspo2-, and Etv1*-positive cells at the level of transcriptomics and spatial expression.

(A) UMAP plots showing *Lypd1-, Rspo2-,* and *Etv1*-positive clusters in BLA.

(B) Expression of Etv1 and Lypd1 in BLA from anterior (left) to posterior (right). The region highlighted with higher magnification is indicated with a white square (Red: BLA^{Etv1}, Green: BLA^{Lypd1} neurons); a cell was assigned positive if the number of puncta was higher than the average number of puncta per cell in the whole BLA section. This was subsequently confirmed by visual inspection by two persons blind to the identity of the probe.
For all images: Cells marked by the empty arrow are positive for the Green channel, cells marked by the arrowhead are positive for the Red channel, and cells marked by the filled arrow are positive for both channels.

(C) Occupancy graphs showing percentages of positive cells expressing one gene or combining two genes, along the anterior-to-posterior axis with representative confocal images (Left: anterior, Right: posterior). Etv1 in Red, Lypd1 in Green.

(D) Expression of Rspo2 and Lypd1 in BLA from anterior (left) to posterior (right) (Red: BLA^{Rspo2}, Green: BLA^{Lypd1} neurons). For details see B.

(E) Occupancy graphs showing percentages of positive cells expressing one gene or combining two genes, along the anterior-to-posterior axis with representative confocal images (Left: anterior, Right: posterior). Rspo2 in Blue, Lypd1 in Green.

(F) Expression of Etv1 and Rspo2 in BLA from anterior (left) to posterior (right) (Red: BLA^{Etv1}, Green: BLA^{Rspo2} neurons). For details see B.

(G) Occupancy graphs showing percentages of positive cells expressing one gene or combining two genes, along the anterior-to-posterior axis with representative confocal images (Left: anterior, Right: posterior). Rspo2 in Blue, Etv1 in Red.

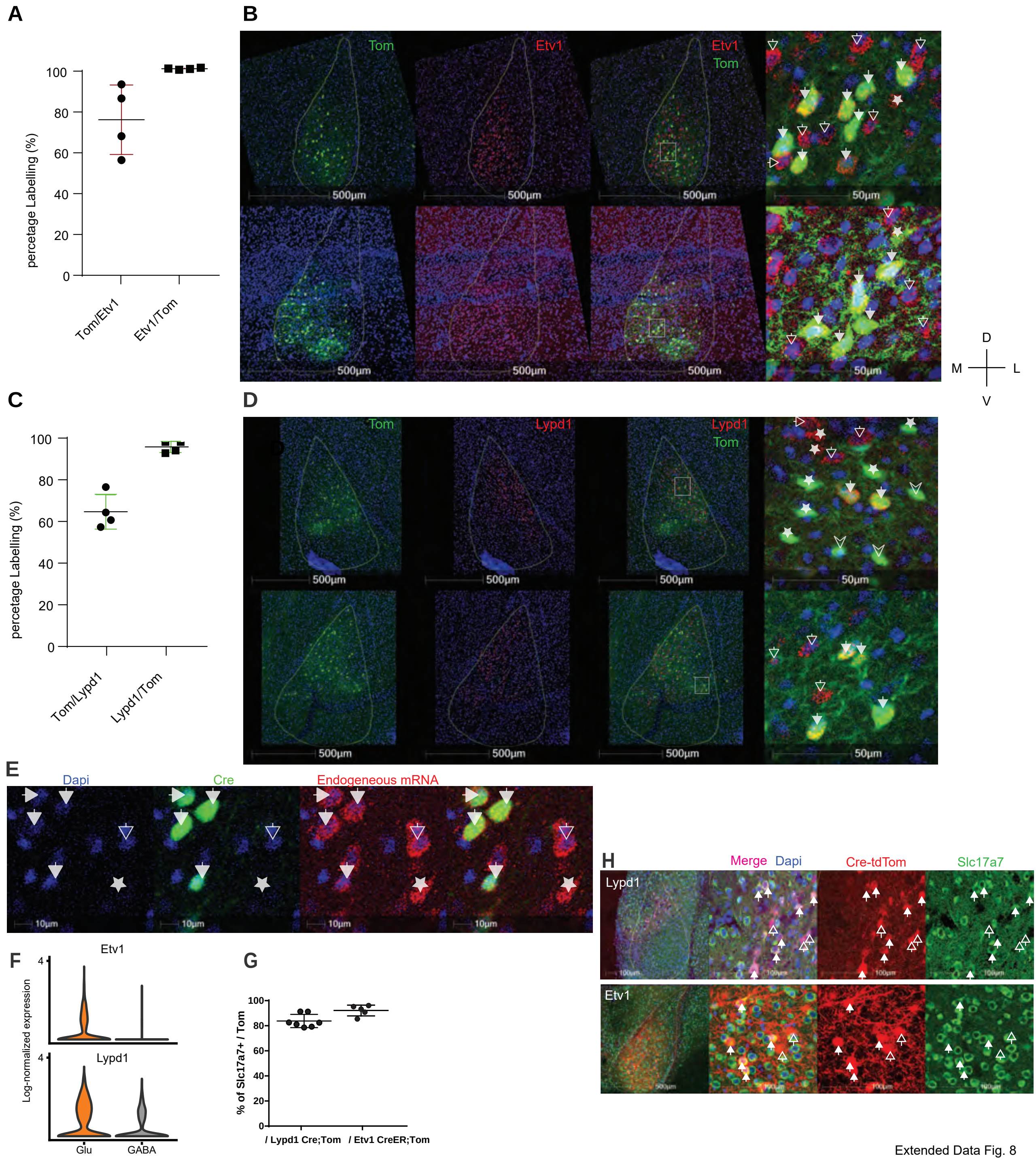

Extended Data Fig. 8

Supplementary Fig. 8 related to Figure 4-7: Validation of Lypd1-Cre and Etv1-Cre lines.

Etv1-CreER and Lypd1-Cre mice were crossed with a Cre-reporter line (Ai9 tdTomato and the expression of tdTomato fluorescence was compared with endogenous Etv1 and Lypd1 mRNA expression by FISH, respectively.

(A) Quantification of the proportion of Cre;Tom cells expressing endogenous Etv1 among all Etv1+ cells (Tom/Etv1) and the percentage of Cre;Tom cells expressing endogenous Etv1 among all Cre;Tom cells (Etv1/Tom) (n = 4 sections from 2 mice).

(B) Example images with Etv1-Cre; tdTomato expression (green) and endogenous Etv1 (red) expression in the BLA (anterior (top) and posterior section (bottom)) with higher-magnified images in each panel (right). Empty arrow-marked cells are positive for Etv1 mRNA signal (Etv1), arrowhead-marked cells are positive for the Cre signal (Tom), and white-filled arrow-marked cells are positive for both Cre and endogenous mRNA signal. Asterisk-marked cells are not considered for quantification due to out of a focal plane without nuclear signal (Dapi).

(C) Quantification of the proportion of Cre;Tom cells expressing endogenous Lypd1 among all Lypd1+ cells (Tom/Lypd1) and the percentage of Cre;Tom cells expressing endogenous Lypd1 among all Cre;Tom cells (Lypd1/Tom) (n = 4 sections from 2 mice).

(D) Example images with Lypd1-Cre; tdTomato expression (green) and endogenous Lypd1 (red) expression in the BLA. (anterior (top) and posterior section (bottom)) with higher-magnified images in each panel (right). Empty arrow-marked cells are positive for Lypd1 mRNA signal (Lypd1), for other details see B.

(E) Explanation of quantification for positive cells for Cre;tdTomato and endogenous expression: white-filled arrow-marked cells are positive for both Cre and endogenous mRNA signal, empty arrow-marked cells are positive only for endogenous mRNA signal, asterisk-marked cells are not considered for quantification due to lack of a nuclear signal (Dapi).

Bar graphs show mean ± SEM, the BLA region is indicated with a thin line in each image.

(F) Violin plots of fractional expression of *Etv1*-expressing cells and *Lypd1*-expressing cells in Glutamatergic cluster or GABAergic cluster from snRNA seq data. The portion of Etv1-expressing cells in the Glutamatergic and GABAergic cluster: 81%, and 19%, respectively. The portion of Lypd1 expressing cells in the Glutamatergic and GABAergic cluster: 71%, and 29%, respectively.

(G) The quantification of the proportion of Lypd1-Cre;Tom cells expressing Slc17a7 among all Lypd1-Cre;Tom cells and the proportion of Etv1-Cre;Tom cells expressing Slc17a7 among all Etv1-Cre;Tom cells: n = 5-7 sections from 2-3 mice.

(H) Representative images for co-labeling of Slc17a7 and Tom in Lypd1 (top) and Etv1 (bottom) Cre;tdTomato groups. The white arrows indicate cells co-expressing Slc17a7 (Slc17a7+) and tdTomato and the empty arrows indicate Tom cells not expressing Slc17a7 ((Slc17a7-).

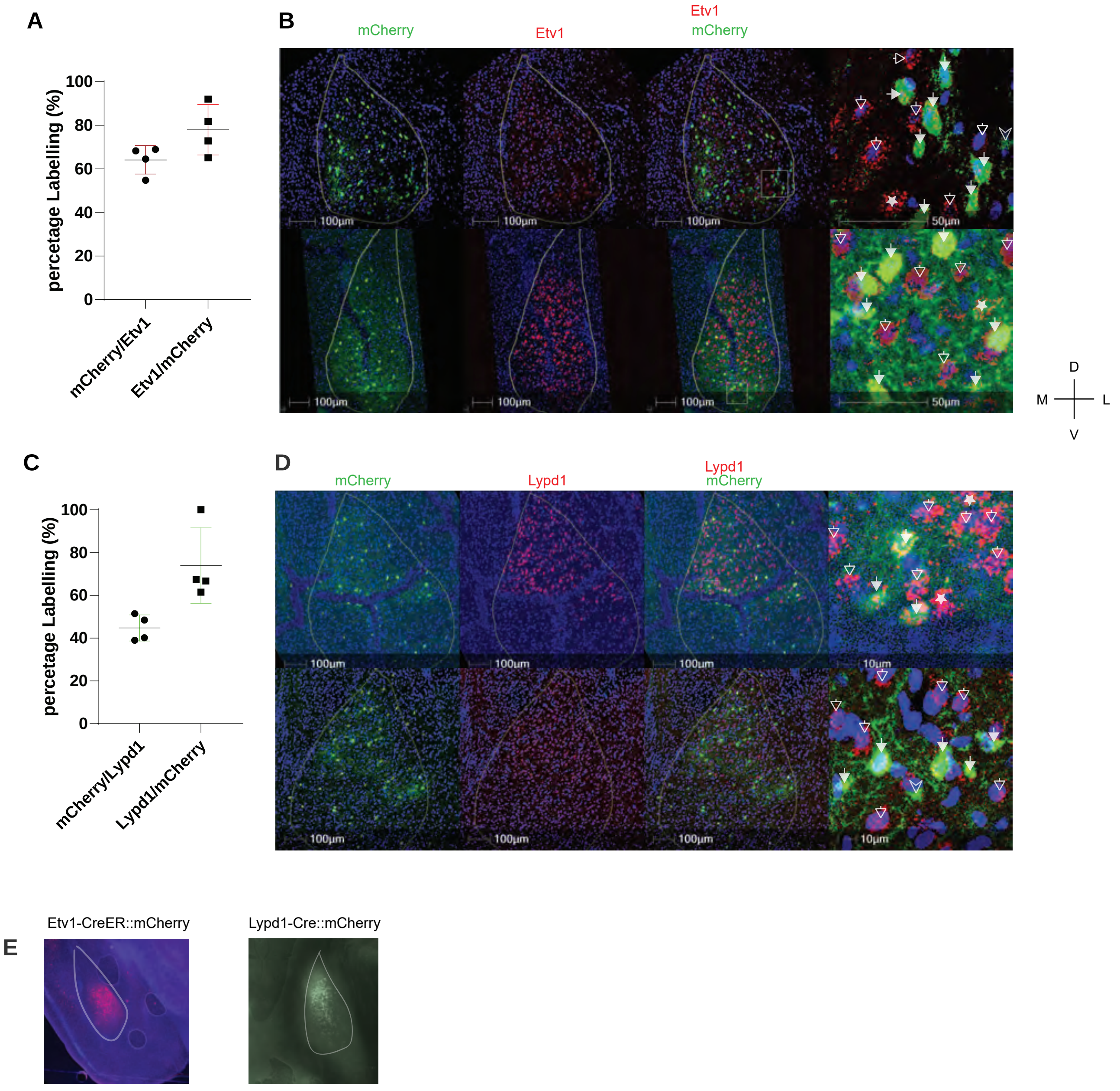
A
percetage Labelling (%)
100
80
60
40
20
0
mCherry/Etv1
Etv1/mCherry
B
mCherry
Etv1
Etv1
mCherry
100µm
50µm
D
M
L
V
C
percetage Labelling (%)
mCherry/Lypd1
Lypd1/mCherry
D
mCherry
Lypd1
Lypd1
mCherry
10µm
E
Etv1-CreER::mCherry
Lypd1-Cre::mCherry

Supplementary Fig. 9 related to Figure 4-7: Validation of Lypd1-Cre and Etv1-Cre lines with viral injection.

Etv1-CreER and Lypd1-Cre mice were injected with a Cre-dependent virus (AAV5-hSyn-DIO-mCherry) and the expression of mCherry fluorescence was compared with endogenous Etv1 and Lypd1 mRNA expression by FISH, respectively.

(A) Quantification of the proportion of Cre::mCherry cells expressing endogenous Etv1 among all Etv1+ cells (mCherry/Etv1) and the percentage of Cre::mCherry cells expressing endogenous Etv1 among all Cre::mCherry cells (Etv1/mCherry) (n = 4 sections from 2 mice).

(B) Example images with Etv1-Cre::mCherry expression (green) and endogenous Etv1 (red) expression in the BLA (anterior (top) and posterior section (bottom)) with higher-magnified images in each panel (right). Empty arrow-marked cells are positive for the Etv1 mRNA signal (Etv1), arrowhead-marked cells are positive for the Cre signal (mCherry), and white-filled arrow-marked cells are positive for both the Cre and endogenous mRNA signal. Asterisk-marked cells are not considered for quantification due to being out of a focal plane without a nuclear signal (Dapi).

(C) Quantification of the proportion of Cre::mCherry cells expressing endogenous Lypd1 among all Lypd1+ cells (mCherry/Lypd1) and the percentage of Cre::mCherry cells expressing endogenous Lypd1 among all Cre::mCherry cells (Lypd1/mCherry) (n = 4 sections from 2 mice).

(D) Example images with Lypd1-Cre::mCherry expression (green) and endogenous Lypd1 (red) expression in the BLA. (anterior (top) and posterior section (bottom)) with higher-magnified images in each panel (right). Empty arrow-marked cells are positive for Lypd1 mRNA signal (Lypd1), for other details see B.

(E) Representative images of mCherry expression in Etv1 and Lypd1-Cre mouse from vibratome (100-150um thickness) cutting slices to confirm on-target expression.

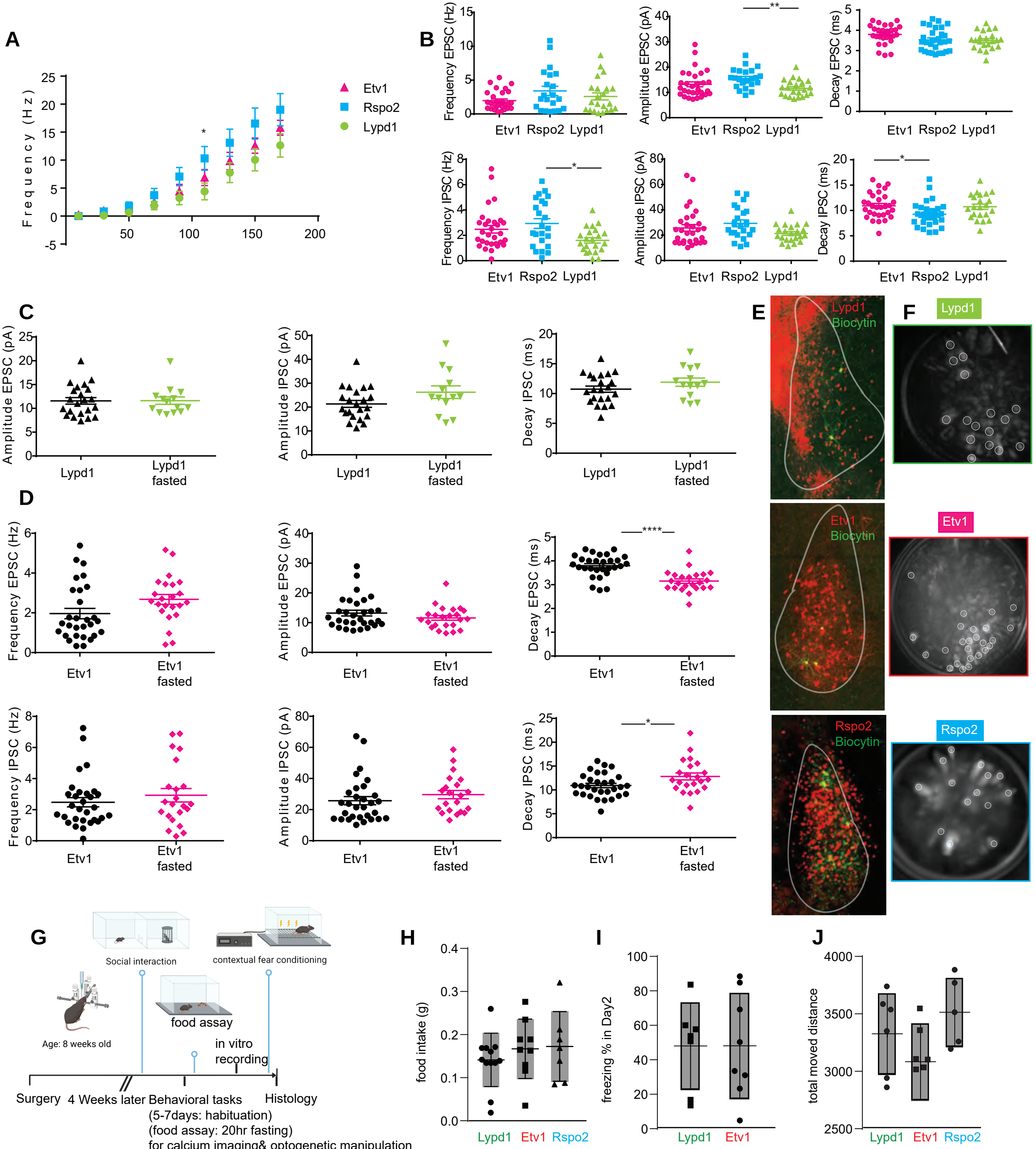

Extended Data Fig. 10

Supplementary Fig. 10 related to Figures 4-5: Electrophysiological properties of three neuronal populations and behavioral parameters during calcium imaging.

(A) Firing rates (Hz) after injecting different current steps in three different types of neurons (colored by types of neurons, Two-way ANOVA Mixed-effects analysis: in Rspo2 vs Lypd1, *p<0.05, marked at the corresponding step. (n= neuron numbers; BLA^{Lypd1} = 22, BLA^{Etv1} = 30 and BLA^{Rspo2} = 21 from 2 mice per group); F (2, 66) = 1.781, P = 0 0.0302.

(B) Synaptic properties: Quantification of frequency, amplitude, decay time (left to right) of sEPSC and sIPSC (top to bottom) in three different neuronal populations. One-way ANOVA, *p<0.05, **p<0.01. (n= neuron numbers; BLA^{Lypd1} = 22, BLA^{Etv1} =30 and BLA^{Rspo2} = 21 from 2 mice per group); (**p=0.0082, *p=0.0136 (Frequency IPSC), *p=0.0252(Decay IPSC).

(C-D) Alterations of synaptic properties after fasting: Quantification of amplitude and decay time of sEPSC and sIPSC in BLA^{Lypd1} neurons in fed or fasted animals (C) and quantification of frequency, amplitude, and decay time of sEPSC (top) and sIPSC (bottom) in BLA^{Etv1}neurons in fed or fasted animals (D). Unpaired t-test, *p<0.05, ****p<0.0001 (Lypd1-fed and-fast group: n = 22, 14 neurons and Etv1-fed and -fasted group: n = 30, 22 neurons from 2 mice per group).

(E) Ex-vivo representative images for neurobiotin-filled BLA ^{Etv1}, BLA ^{Lypd1} and BLA ^{Rspo2} neurons after whole cell patch-clamping in Etv1-Cre;tdTomato, Lypd1-Cre; tdTomato and Rspo2-Cre; tdTomato mouse, respectively. (tdTomato + neurons (Red), Ex-vivo biocytin neurons (Green) and overlapping neurons (Yellow)).

(F) Representative maximum-projection images of focal planes of BLA ^{Etv1}, BLA ^{Lypd1} and BLA ^{Rspo2} -GCaMP6f expressing neurons. White circles indicated selected ROIs.

(G)Timeline of behavioral tests for calcium imaging and optogenetic manipulation, created with BioRender.com released under a Creative Commons Attribution-NonCommercial-NoDerivs 4.0 International license.

(H)Average of food consumption in the free-feeding assay during calcium imaging (600s): Lypd1 group: n= 12, mean =0.1395, std=0.06151. Etv1 group: n=9, mean = 0.1664, std= 0.06972. Rspo2 group= n=7, mean =0.1718, std=0.08155 (Dunn's multiple comparisons test, ns; P=0.5770).

(I) Average of freezing time (%) on Day 2 during CFC calcium imaging (180s): Lypd1 group: n= 7, mean = 48.06, std= 24.86. Etv1 group: n=8, mean = 48.17, std= 30.29 (Kolmogorov-Smirnov test, ns;p= 0.6601).

(J)Average of total distance moved (cm) in the social assay during calcium imaging (600s): Lypd1 group: mean= 3323.64 Std= 357.206, n=6, Etv1 group: mean= 3072.7, Std= 332.517, n= 7, Rspo2 group: mean= 3505.542, Std= 300.4131387, n= 5 (Tukey's multiple comparisons test, ns; $F(2, 15) = 2.527$ $P=0.1133$).

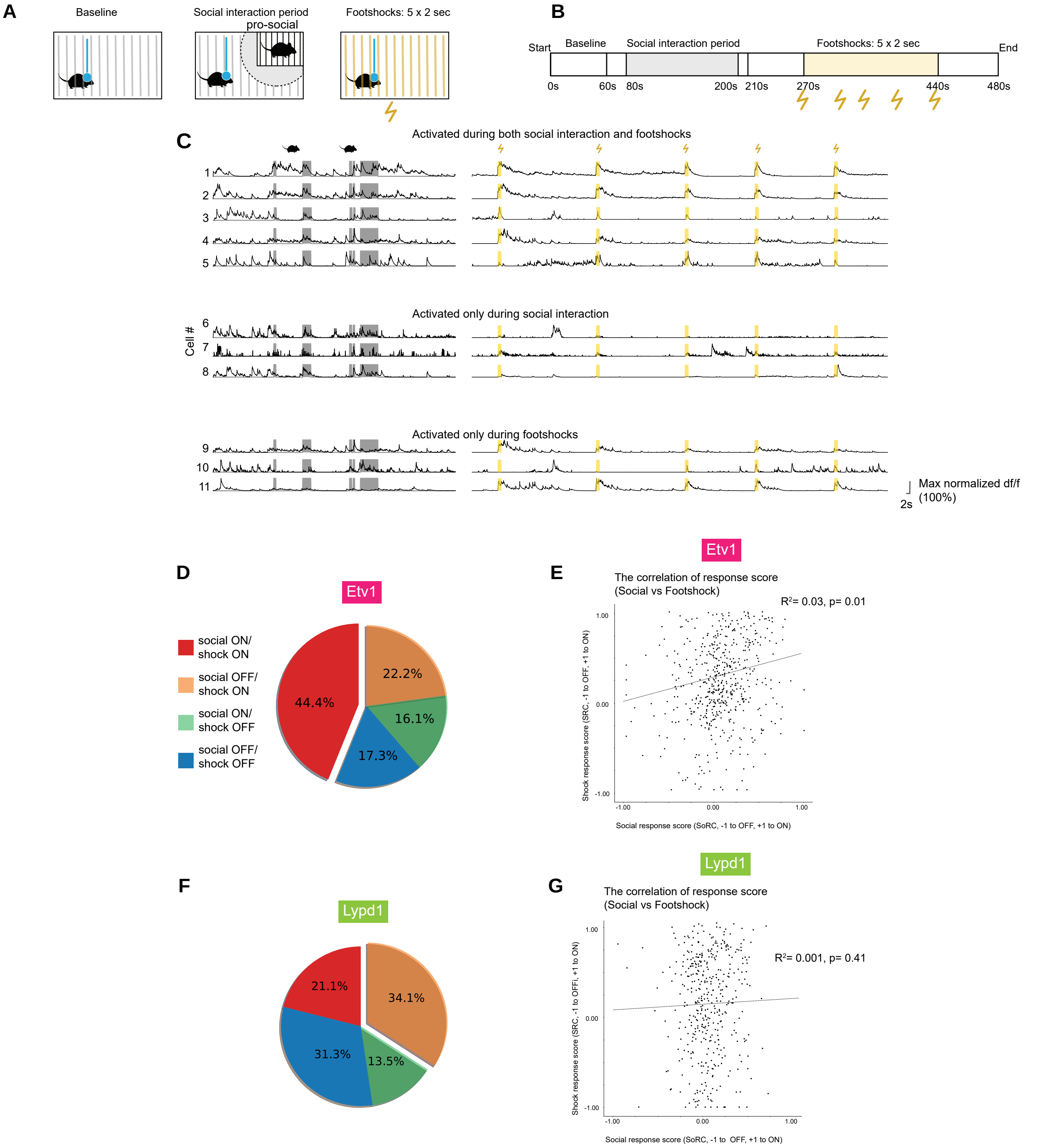

Extended Data Fig. 11

Supplementary Fig. 11 related to Figure 4-5: Mixed selectivity of BLA^{Etv1} neurons for social interaction and footshock responses.

(A) Scheme of social interaction and footshock assay (Width:17cm, Length: 24cm).

(B) Behavioral protocol for calcium imaging during social interaction and footshocks in the same session: 0s-60s, Baseline imaging of an experimental mouse freely exploring the CFC chamber without a social conspecific; 60s-80s, introduction of a social conspecific confined in a wired container; 80s-200s, social interaction period; 200s-210s, removal of wired container; 270s- 440s, five footshocks at 270s, 320s, 365s, 400s, and 440s.

(C) Representative calcium traces of BLA^{Etv1} neurons that showed increased activity during social interaction and footshocks (top), only during social interaction (middle), and only during footshocks (bottom). Social interaction and footshock bouts are shaded in gray and yellow, respectively.

(D) Fractions of BLA^{Etv1} neurons that were activated during social interactions (social ON) and/or footshocks (shock ON) or remained inactive (social OFF and/or shock OFF) as indicated. Percentage values are indicated in the pie chart.

(E) Distribution of SoRCs and SRCs of BLA^{Etv1} neurons (n=481 from 4 animals). The black line indicates the linear model fitted to the data. The R^2 and p-values for the linear models are indicated on the plots.

(F) Fractions of BLA^{Lypd1} neurons that were activated during social interactions (social ON) and/or footshocks (shock ON) or remained inactive (social OFF and/or shock OFF) as indicated. Percentage values are indicated in the pie chart.

(G) Distribution of SoRCs and SRCs of BLA^{Lypd1} neurons (n = 422 from 3 animals). The black line indicates the linear model fitted to the data. The R^2 and p-values for the linear models are indicated on the plots.

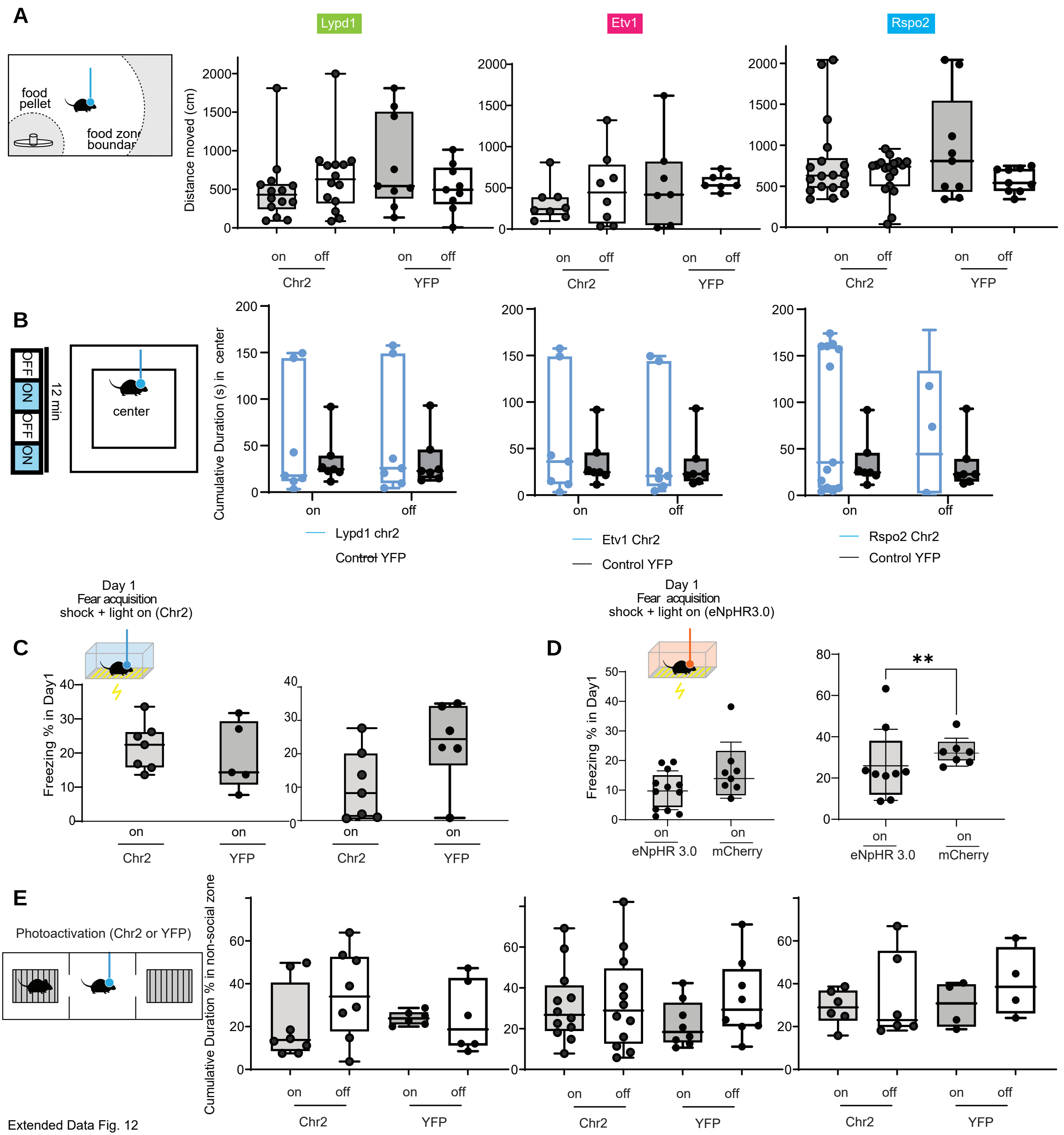

Extended Data Fig. 12

Supplementary Fig. 12 related to Figures 6-7: Motor, non-social and anxiety-related behaviors during optogenetic manipulations.

(A) Left: Scheme of free-feeding assay. Right: Total distance travelled in free-feeding assay combined with photoactivation (related to Figure 6C). Lypd1 group; n = 14 (ChR2) and 9 mice (YFP) per group with one-way ANOVA (Kruskal-Wallis test): p= 0.4126). Etv1 group; n = 8 (ChR2) and 7 mice (YFP) per group with one-way ANOVA (Kruskal-Wallis test): p= 0.2647). Rspo2 group; n = 18 (ChR2) and 9 (YFP) per group with one-way ANOVA (Kruskal-Wallis test), p= 0.5058).

(B) Left: Scheme of Open-field test (OFT) with light on (ON) and light off (OFF) illumination epochs. Right: cumulative duration (s) in center zone per 3 min of each epoch. Lypd1 groups; n = 7 mice in each ChR2 and YFP groups with two-way ANOVA group × epoch interaction (F (1, 24) = 0.003498, p=0.9533; Bonferroni *post hoc* analysis). Etv1 groups; n = 7 mice in each ChR2 and YFP groups with two-way ANOVA group × epoch interaction (F (1, 24) = 0.05877, p=0.8105; Bonferroni *post hoc* analysis). Rspo2 groups; n = 13 (ChR2) and 7 mice (YFP) per group with two-way ANOVA group × epoch interaction (F (1, 36) = 0.2086, p=0.6506; Tukey's multiple comparisons test, Bonferroni *post hoc* analysis). We only considered each 2nd ON and 2nd OFF epoch in order to avoid other behavioral contaminations.

(C) Freezing behavior (%) on Day 1 during CFC combined with photoactivation of BLA^{Lypd1} or BLA^{Etv1} neurons. The percentage of freezing was analyzed during 300s after 1st footshock offset: Lypd1 groups; n =7 (ChR2) and 5 mice (YFP) per group with Kolmogorov-Smirnov test, ns (p= 0.4343). Etv1 groups; n = 6 (ChR2) and 7 mice (YFP) per group with Kolmogorov-Smirnov test, ns (p= 0.0676).

(D) Freezing behavior (%) on Day 1 during CFC combined with photoinhibition of BLA^{Lypd1} or BLA^{Etv1} neurons. The percentage of freezing was analyzed during 300s after 1st footshock offset: Lypd1 group; n = 12 mice and 8 mice per eNpHR 3.0 and mcherry group, respectively with Kolmogorov-Smirnov test, p= 0.5095. Etv1 group; n = 9, 7 mice per each eNpHR 3.0 and mcherry group, respectively with Kolmogorov-Smirnov test, p = 0.0052, **p<0.01.

(E) Left: Scheme of social interaction assay. Right: Cumulative duration (%) in non-social zone combined with photoactivation of three BLA populations in the social assay in comparison to light-off epochs and YFP controls. Lypd1 group; n = 8 (ChR2) and 6 mice (YFP) per group with one-way ANOVA (Kruskal-Wallis test; Bonferroni *post hoc* analysis), ns (p= 0.2574). Etv1 group; n = 12 (ChR2) and 8 mice (YFP) per group with one-way ANOVA (Kruskal-Wallis test; Bonferroni *post hoc* analysis), ns (p=0.0407). Rspo2 group; n = 6 (ChR2) 4 mice (YFP) per

group with one-way ANOVA ((Kruskal-Wallis test; Bonferroni *post hoc* analysis), ns (p= 0.6801).

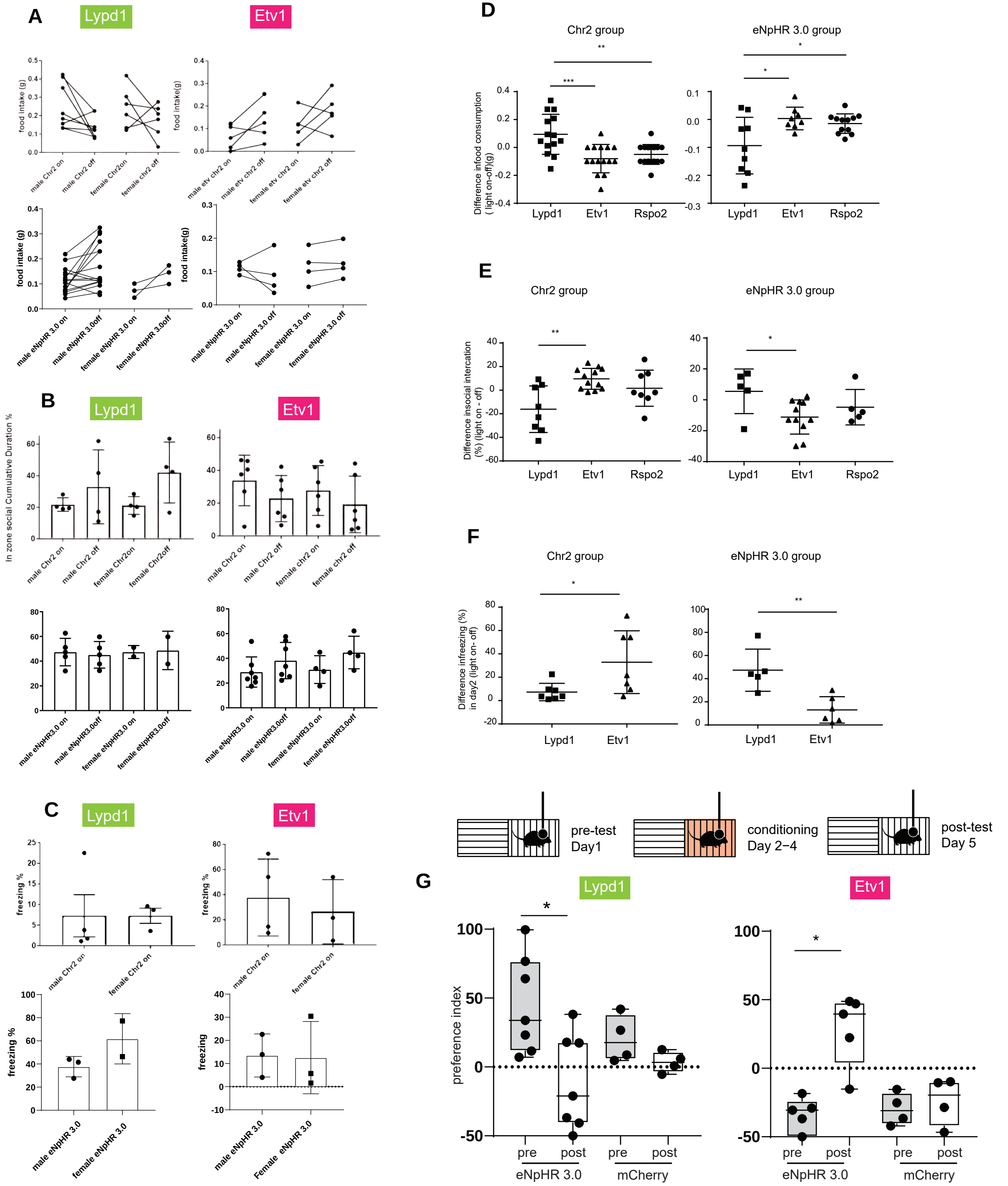

Extended Data Fig. 13

Supplementary Fig. 13 related to Figures 6-7: No sex differences.

(A) Food intake during optogenetic manipulation of BLA^{Lypd1} and BLA^{Etv1} populations compared to light-off epochs in male and female mice. ChR2 groups (top): Lypd1 group, Male: n=8, Female: n=6 with F (3, 24) = 2.301, P=0.1028, Etv1 group: Male: n=6, Female: n=5 with F (3, 18) = 2.201, P=0.1231; Tukey's multiple comparisons test, Bonferroni post hoc analysis. eNpHR 3.0 groups (bottom):Lypd1 group, Male: n=7, Female: n=3 with F (3, 34) = 1.879, P=0.0598, Etv1 group: Male: n=4, Female: n=4 with F (3, 12) = 0. 7522, P=0. 7624; Tukey's multiple comparisons test.

(B) Cumulative duration in the social zone (%) during optogenetic manipulation of BLA^{Lypd1} and BLA^{Etv1} populations compared to light-off epochs in male and female mice. ChR2 groups (top): Lypd1 group: Male: n=4, Female: n=4 with F (3, 12) = 1.647, P=0.2309; Etv1 group: Male: n=6, Female: n=6 with F (3, 20) = 0.9957, P=0.4151 Tukey's multiple comparisons test, Bonferroni post hoc analysis. eNpHR 3.0 groups (bottom): Lypd1 group, Male: n=5, Female: n=2 with F (3, 10)= 0. 5327, P=0.0598, Etv1 group: Male: n=8, Female: n=4 with F (3, 18) = 0. 2474, P=0. 3694; Tukey's multiple comparisons test.

(C) Freezing behavior (%) on day 2 combined with photo-manipulation of BLA^{Lypd1} and BLA^{Etv1} populations in males and females. ChR2 groups (top Lypd1 group: Male: n=4, Female: n=3 with P= 0.9989, Etv1 group: Male: n=4, Female: n=3 with P= 0.6284; eNpHR 3.0 groups (bottom): Lypd1 group, Male: n=3, Female: n=2 with P= 0.1666, Etv1 group: Male: n=3 Female: n=3 with P=0. 9327; Unpaired t-test.

(D-E) Data were generated from Fig. 6-7.

(D) Differences in food consumption across three mouse lines. Left; mice from ChR2 groups across Lypd1, Etv1, and Rspo2 lines. Lypd1: 14, Etv1: 15, Rspo2: 18 mice; ChR2 on Lypd1 vs. ChR2 on Etv1: adjusted p= 0.0002, ChR2 on Lypd1 vs. ChR2 on Rspo2: adjusted p= 0.0012, ChR2 on Etv1 vs. ChR2 on Rspo2: adjusted p= 0.7006, Tukey's multiple comparisons test, Bonferroni post hoc analysis. Right: mice from eNpHR 3.0 groups across Lypd1, Etv1, and Rspo2 lines. Lypd1: 9, Etv1: 8, Rspo2: 12mice, Lypd1 vs. Etv1: adjusted p= 0.0119, Lypd1 vs. Rspo2: adjusted p=0.0256, Etv1 vs. Rspo2:

adjusted p= 0.8077. Tukey's multiple comparisons test, Bonferroni post hoc analysis. *p<0.05, **p<0.01, ***p<0.001.

(E) Differences in social interaction across three mouse lines. Left; mice from ChR2 groups across Lypd1, Etv1, and Rspo2 lines. Lypd1: 8, Etv1:12, Rspo2: 8 mice, ChR2 on Lypd1 vs. ChR2 on Etv1: adjusted p= 0.0017, ChR2 on Lypd1 vs. ChR2 on Rspo2: adjusted p=0.0533, ChR2 on Etv1 vs. ChR2 on Rspo2: adjusted p=0.456. Tukey's multiple comparisons test, Bonferroni post hoc analysis. Right: mice from eNpHR 3.0 groups across Lypd1, Etv1, and Rspo2 lines. Lypd1: 5, Etv1: 11, Rspo2: 5mice, Lypd1 vs. Etv1: adjusted p=0.0474, Lypd1 vs. Rspo2: adjusted p=0.3832, Etv1 vs. Rspo2= adjusted p=0.5939. Tukey's multiple comparisons test, Bonferroni post hoc analysis. *p<0.05, **p<0.01, ***p<0.001.

(F) Differences in freezing on fear conditioning Day 2 across two mouse lines. Left: mice from ChR2 groups across Lypd1, Etv1 lines. Lypd1: 7, Etv1: 8 mice, P= 0.0320 with two-tailed unpaired t-test. Right: mice from eNpHR 3.0 groups across Lypd1, Etv1 lines. Lypd1:5, Etv1: 6 mice, P= 0.0041 with two-tailed unpaired t-test. *p<0.05, **p<0.01, ***p<0.001.

(G) Left: Scheme of conditioned-place preference experiment. Right: Preference index (cumulative time % in paired chamber – cumulative time % in unpaired chamber) of cohorts of mice combined with photoinhibition of two BLA populations before (pre) and after (post) conditioning. In the case of Lypd1-Cre mice, the initially preferred chamber was paired with light, in the case of Etv1-Cre mice, the initially non-preferred chamber was paired with light, to observe avoidance and preference, respectively. Lypd1 groups: n = 7 (eNpHR 3.0) and 4 mice (mCherry) per group; two-tailed paired t-test, $t_{(6)}$ = 2.664, p= 0.0373 within eNpHR 3.0 group (pretest versus posttest) and ns, $t_{(3)}$ =1.484, p= 0.2345 within mCherry group. Etv1 groups: n = 5 (eNpHR 3.0) and 4 mice (mCherry) per group; two-tailed paired t-test, $t_{(4)}$ =3.418, p = 0.0268 within eNpHR 3.0 group (pretest versus posttest) and ns, $t_{(3)}$ =1.466, p= 0.2388 within mCherry group. *p<0.05, **p<0.01, ***p<0.001.

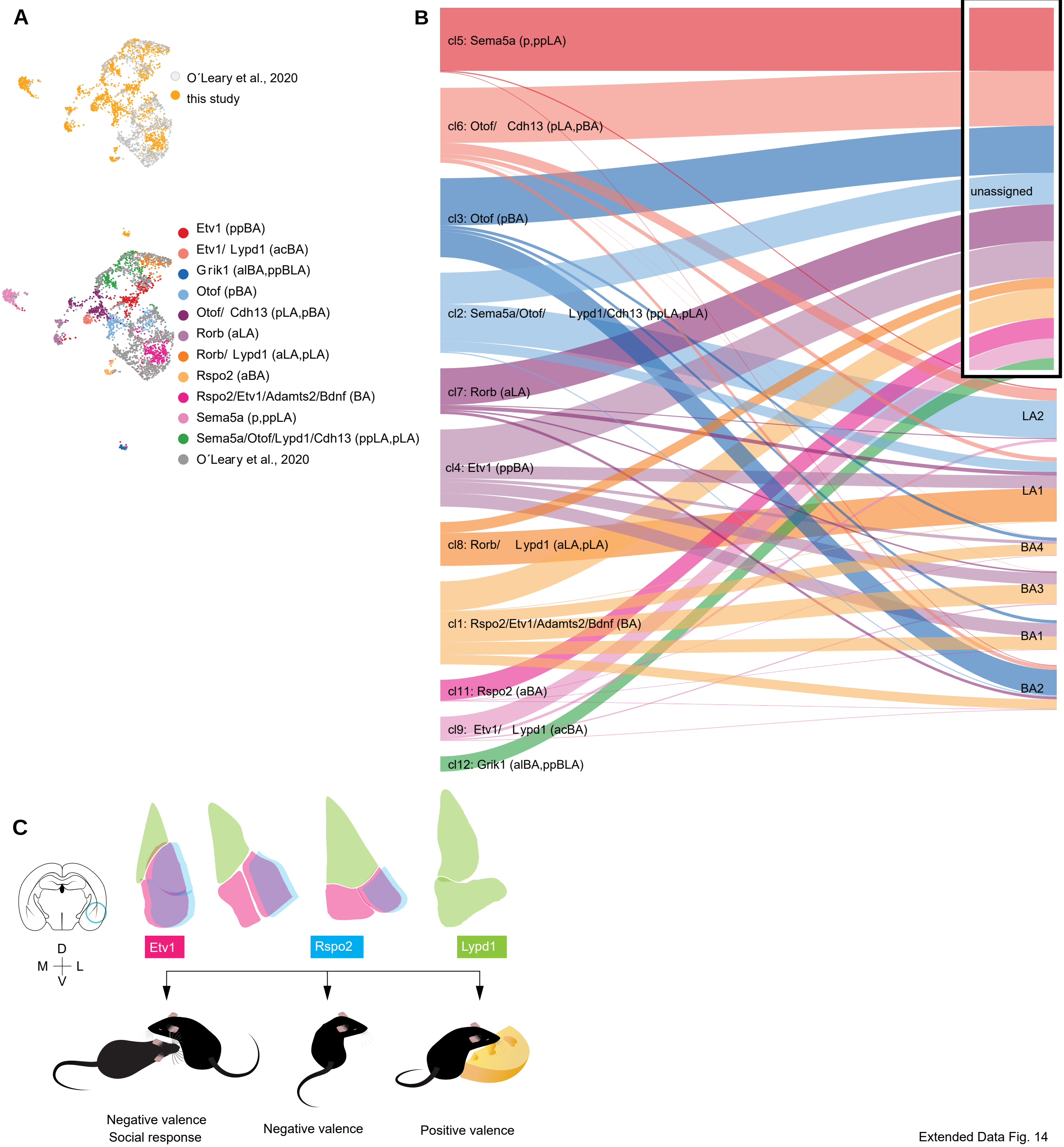

Extended Data Fig. 14

Supplementary Fig. 14 related to discussion: Comparison with published scRNAseq data and graphical summary of our findings.

(A) UMAP of glutamatergic BLA neurons from published data [4] aligned with our glutamatergic BLA clusters (top: cells aligned from both datasets; published data (grey) and our data (yellow), bottom: clusters of our dataset in different colors, published data in grey).

(B) River plots showing the match flow from our data (left) to published data (right); Unique cell clusters in our data without corresponding cluster in published data indicated as "unassigned". The thickness of lines represents the number of cells matching cluster assignments.

(C) Schematic drawing of three different neuronal types in BLA with their distinct spatial expression pattern across the anterior-posterior extent of the BLA (from left to right) and encoding positive, negative, or mixed negative/social valence.

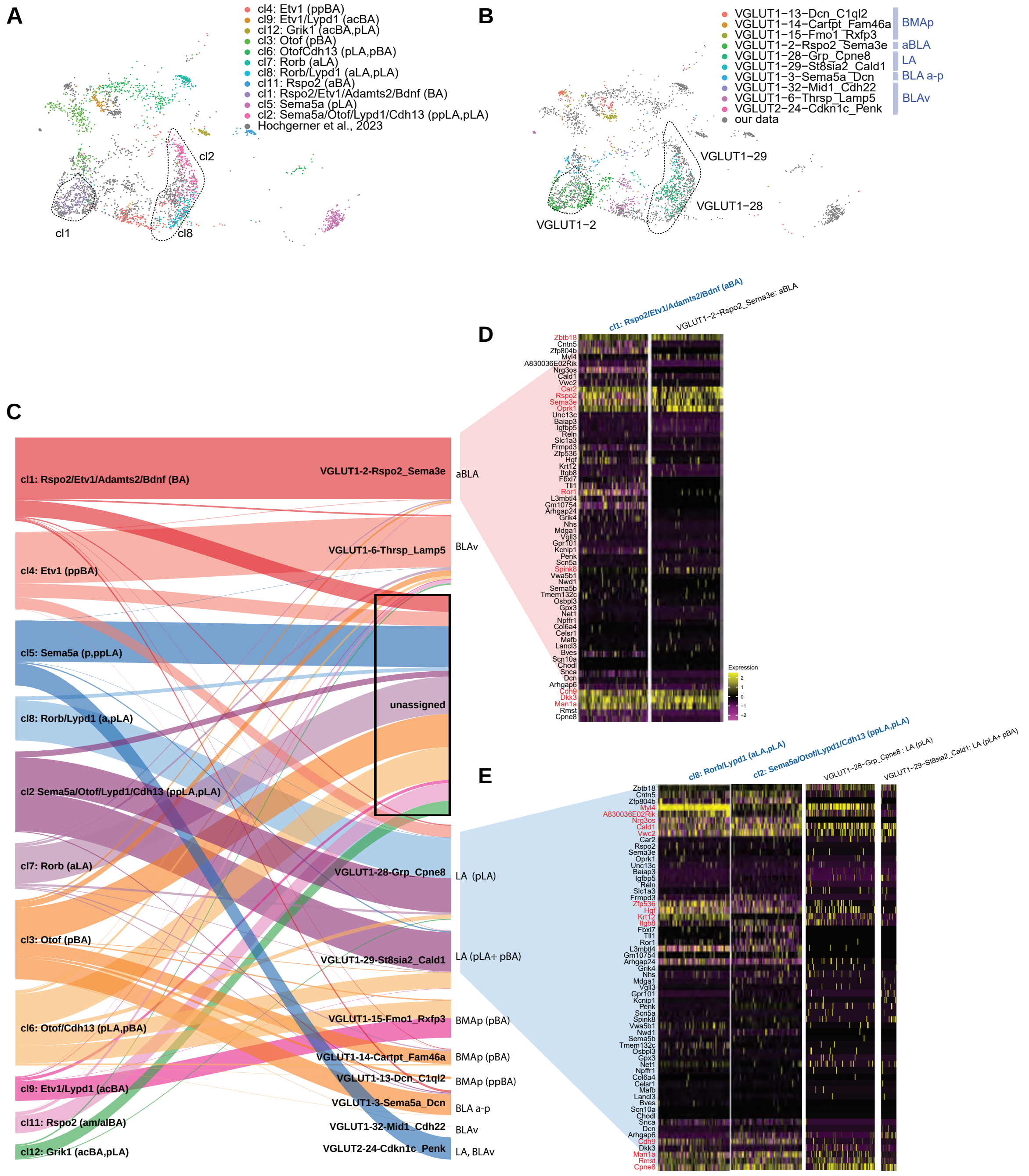

Extended Data Fig. 15

Supplementary Fig. 15 related to discussion: Comparison with another published scRNAseq data.

(A) UMAP of glutamatergic BLA neurons from another published (Hochgerner et al) data [3] aligned with our glutamatergic BLA clusters (BLA cells from Hochgerner's data (grey) and our data (color-coded each cluster)). Clusters marked by dashed lines showed a good match between the two datasets.

(B) UMAP of ten glutamatergic BLA clusters from Hochgerner's data (color-coded clusters) aligned with our data (Gray) and the anatomical annotation for each Hochgerner's cluster (Blue). Clusters marked by dashed lines showed a good match between the two datasets.

(C) River plots showing the match flow from our data (left) to published data (right); Unique cell clusters in our data without corresponding clusters in published data indicated as "unassigned". The thickness of lines represents the number of cells matching cluster assignments. Anatomical annotations from the Hochgerner study are indicated on the right and our modified suggestions are written in brackets.

(D) Heatmap of genes co-expressed in the integrated datasets between cl1: Rspo2/Etv1/Adamts2/Bdnf (aBA) and VGLUT1-2; Rspo2_Sema3e (aBLA). Red-colored genes are highly expressed in both clusters. The clusters from this study are written in blue and the clusters from the Hochgerner dataset are written in black font. The average gene expression level by color scale.

(E) Heatmap of genes co-expressed in the integrated datasets between cl8: Rorb/Lypd1 (aLA,pLA), cl2: Sema5a/Otof/Lypd1/Cdh13 (ppLA,pLA) and VGLUT1−29−St8sia2_Cald1: LA (pLA+ pBA), VGLUT1−28−Grp_Cpne8: LA (pLA). Red-colored genes are highly expressed in both clusters. The clusters from this study are written in blue and the clusters from the Hochgerner dataset are written in black font. The average gene expression level by color scale.

References

1. Hintiryan, H. *et al.* Connectivity characterization of the mouse basolateral amygdalar complex. *Nat Commun* **12**, 2859 (2021).

2. Kim, J., Pignatelli, M., Xu, S., Itohara, S. & Tonegawa, S. Antagonistic negative and positive neurons of the basolateral amygdala. *Nat Neurosci* **19**, 1636–1646 (2016).

3. Kim, J., Zhang, X., Muralidhar, S., LeBlanc, S. A. & Tonegawa, S. Basolateral to Central Amygdala Neural Circuits for Appetitive Behaviors. *Neuron* **93**, 1464-1479.e5 (2017).

4. O'Leary, T. P. *et al.* Extensive and spatially variable within-cell-type heterogeneity across the basolateral amygdala. *Elife* **9**, e59003 (2020).

5. Hochgerner, H. *et al.* Neuronal types in the mouse amygdala and their transcriptional response to fear conditioning. *Nat Neurosci* **26**, 2237–2249 (2023).
