# Supplementary material for: Genetically- and spatially-defined basolateral amygdala neurons control food consumption and social interaction
Source: Nat Commun. 2024 Aug 11;15:6868. doi: 10.1038/s41467-024-50889-7 (PMC11316773; doi:10.1038/s41467-024-50889-7)
Supplement: Supplementary file 3 — Reporting Summary [file 41467_2024_50889_MOESM3_ESM.pdf]

Corresponding author(s): Ruediger Klein

Last updated by author(s): Jun 20, 2024

Reporting Summary

Nature Portfolio wishes to improve the reproducibility of the work that we publish. This form provides structure for consistency and transparency in reporting. For further information on Nature Portfolio policies, see our Editorial Policies and the Editorial Policy Checklist.

Statistics

For all statistical analyses, confirm that the following items are present in the figure legend, table legend, main text, or Methods section.

n/a	Confirmed	
☐	☒	The exact sample size (*n*) for each experimental group/condition, given as a discrete number and unit of measurement
☐	☒	A statement on whether measurements were taken from distinct samples or whether the same sample was measured repeatedly
☐	☒	The statistical test(s) used AND whether they are one- or two-sided<br>*Only common tests should be described solely by name; describe more complex techniques in the Methods section.*
☒	☐	A description of all covariates tested
☐	☒	A description of any assumptions or corrections, such as tests of normality and adjustment for multiple comparisons
☐	☒	A full description of the statistical parameters including central tendency (e.g. means) or other basic estimates (e.g. regression coefficient) AND variation (e.g. standard deviation) or associated estimates of uncertainty (e.g. confidence intervals)
☐	☒	For null hypothesis testing, the test statistic (e.g. *F*, *t*, *r*) with confidence intervals, effect sizes, degrees of freedom and *P* value noted<br>*Give P values as exact values whenever suitable.*
☒	☐	For Bayesian analysis, information on the choice of priors and Markov chain Monte Carlo settings
☒	☐	For hierarchical and complex designs, identification of the appropriate level for tests and full reporting of outcomes
☐	☒	Estimates of effect sizes (e.g. Cohen's *d*, Pearson's *r*), indicating how they were calculated

Our web collection on statistics for biologists contains articles on many of the points above.

Software and code

Policy information about availability of computer code

Data collection	epifluorescence microscope (Zeiss) with 10× or 5x/0.3 objectives (Zeiss)<br>Leica SP8 confocal microscope equipped with a 20×/0.75 IMM objective (Leica)<br>nVista HD 2.1 (Inscopix)<br>Radiant 2.0 (Plexon)<br>pClamp 10 (Molecular Devices)
Data analysis	Clampex 10.3 and Clampfit (Molecular Devices)<br>ImageJ 2.0.0-rc-49/1.51a (NIH)<br>Prism 7 (GraphPad Software)<br>R (Version 0.98.1103)<br>Ethovision XT 16 (Noldus)<br>Python (3.10.8)<br>HALO software (Indica Labs)<br>IDPS (Inscopix)

For manuscripts utilizing custom algorithms or software that are central to the research but not yet described in published literature, software must be made available to editors and reviewers. We strongly encourage code deposition in a community repository (e.g. GitHub). See the Nature Portfolio guidelines for submitting code & software for further information.

Data

Policy information about availability of data

All manuscripts must include a data availability statement. This statement should provide the following information, where applicable:

- Accession codes, unique identifiers, or web links for publicly available datasets
- A description of any restrictions on data availability
- For clinical datasets or third party data, please ensure that the statement adheres to our policy

The raw data for electrophysiology (Fig4), Calcium imaging (Fig4-5) and optogenetic (Fig6-7) have been deposited in the google drive: https://drive.google.com/drive/folders/166KtcgxC-Ad27MZH-lidATxEQKgC0BMf

Raw and processed snRNAseq data are available at GEO (accession number GSE244860).

Also, Custom-written code for calcium imaging (Fig 4-5) is publicly available in a GitHub repository at https://github.com/limserenahansol/1p_BLA_sync_permutation_social_valence
Or in Zenodo: https://doi.org/10.5281/zenodo.11995740

Research involving human participants, their data, or biological material

Policy information about studies with human participants or human data. See also policy information about sex, gender (identity/presentation), and sexual orientation and race, ethnicity and racism.

Reporting on sex and gender	n/a
Reporting on race, ethnicity, or other socially relevant groupings	n/a
Population characteristics	n/a
Recruitment	n/a
Ethics oversight	n/a

Note that full information on the approval of the study protocol must also be provided in the manuscript.

Field-specific reporting

Please select the one below that is the best fit for your research. If you are not sure, read the appropriate sections before making your selection.

☒ Life sciences ☐ Behavioural & social sciences ☐ Ecological, evolutionary & environmental sciences

For a reference copy of the document with all sections, see nature.com/documents/nr-reporting-summary-flat.pdf

Life sciences study design

All studies must disclose on these points even when the disclosure is negative.

Sample size	No statistical methods were used to predetermine sample size. The sample sizes were chosen based on published studies in the field. For respective references, see Statistics paragraph in Methods section.
Data exclusions	All exclusion criteria were established prior to data collection:<br>snRNA-seq: We included high-quality cells only after quality control; a) more than 200 genes detected, b) less than 20% mitochondrial gene reads, c) more than 5% ribosomal protein gene reads, d) less than 0.2% hemoglobin gene reads, e) singlets ; see Methods<br>For deep brain imaging experiments, mice were post-hoc excluded from the analysis if the GRIN lens was placed outside of the basolateral amygdala (BLA) or the virus failed to express.<br>For optogenetic manipulation experiments, animals were excluded from the analysis if (1) they did not show bilateral expression of the virus in the BLA, (2) major virus expression was detected outside of the BLA or (3) they did not exhibit correct fiber placement.<br>For slice electrophysiology, cells were excluded from the analysis if post-hoc the recorded biocytin-filled cell was negative for tdTomato reporter fluorescence.
Replication	All attempts at replication were successful.<br>smFISH: All smFISH experiments were carried out using a minimum of 4 relevant sections per each of eight sub-regions from 6 mice. The exact numbers of repetitions (individual data points from separate tissue sections) were provided in excel files in the raw data repository. Averaging across multiple samples was indicated where applicable and the sample sizes were given.

All protocols, reagents and mouse lines used for the experiments are described in detail in the Methods section and supplementary figures. Further information can be requested from the corresponding author to ensure that our findings can be replicated in other laboratories.

Randomization	Littermates were randomly allocated to experimental groups without pre-determined criteria and could be later identified by unique markers for group assignment.
Blinding	Experiments were conducted by an investigator with knowledge of the animal genotype and treatment. For behavioral experiments, post-hoc verification of virus expression and optic fiber placement were double checked by someone without knowledge of the behavioral data to ensure that data collection was unbiased.

Reporting for specific materials, systems and methods

We require information from authors about some types of materials, experimental systems and methods used in many studies. Here, indicate whether each material, system or method listed is relevant to your study. If you are not sure if a list item applies to your research, read the appropriate section before selecting a response.

Materials & experimental systems

n/a	Involved in the study	
☐	☒	Antibodies
☒	☐	Eukaryotic cell lines
☒	☐	Palaeontology and archaeology
☐	☒	Animals and other organisms
☒	☐	Clinical data
☒	☐	Dual use research of concern
☒	☐	Plants

Methods

n/a	Involved in the study	
☒	☐	ChIP-seq
☒	☐	Flow cytometry
☒	☐	MRI-based neuroimaging

Antibodies

Antibodies used	Chicken polyclonal anti-GFP Thermofischer scientific Cat#A10262 and anti-Slc17a7 (48-2400, Invitrogen).<br>streptavidin conjugated to Alexa Fluor 405 (Thermo Fisher Scientific, S32351, Lot# 1712187). Dilutions and protocols are indicated in the respective Methods sections.
Validation	All antibodies are widely used and commercially available.

Animals and other research organisms

Policy information about studies involving animals; ARRIVE guidelines recommended for reporting animal research, and Sex and Gender in Research

Laboratory animals	Mus musculus, transgenic lines:<br>Rspo2-Cre transgenic line (C57BL/6J-Tg(Rspo2-cre)BIto (RBRC10754)) from RIKEN BioResource Research Center (https://web.brc.riken.jp) and Etv1-CreER transgenic line (Etv1tm1.1(cre/ERT2)) from Jackson Laboratory (www.jax.org/jaxmice) and Lypd1-Cre (Tg(Lypd1-cre)SE5Gsat/Mmucd) mice were imported from the Mutant Mouse Regional Resource Center (https://www.mmrrc.org/).<br>All transgenic mouse lines were backcrossed to C57BL/6J (Jackson Laboratory #000664) background.<br>Age: 2-5 months for behavioral experiments, P56 for snRNA and smFISH data.<br>Animals used for optogenetic manipulations and calcium imaging were handled and singly housed (calcium imaging) or littermate-caged (optogenetic) on a 12 h inverted light cycle for at least 5 days before the experiments. Mice were given ad libitum food access except during food deprivation for feeding experiments. All behavioral assays were conducted at a consistent time during the dark period (2 p.m.–7 p.m.).
Wild animals	none
Reporting on sex	Both males and females were used
Field-collected samples	none
Ethics oversight	All the experiments were performed following regulations from the government of Upper Bavaria. License number ROB-55.2-2532.Vet_02-22-16

Note that full information on the approval of the study protocol must also be provided in the manuscript.

Plants

Seed stocks	n/a
Novel plant genotypes	n/a
Authentication	n/a
